# Deafness, Hearing Loss and the Development of Mental State Reasoning Skills: A Review

**DOI:** 10.1162/opmi.a.1

**Published:** 2025-06-25

**Authors:** Marek Meristo, Luca Surian

**Affiliations:** Department of Psychology, University of Gothenburg, Gothenburg, Sweden; Department of Psychology and Cognitive Science, University of Trento, Trento, Italy

**Keywords:** social cognition, deafness, cognitive development, theory of mind

## Abstract

Individuals with hearing loss have a diverse spectrum of auditory experiences, shaped by the degree of hearing loss and interventions. The study of social cognition in deaf children and more generally, children with hearing loss, contributes to a nuanced understanding of how learning experiences influence social and cognitive development. Research suggests that limited access to language may influence conceptual development in theory of mind or the development of information processing skills required in mental state reasoning. In this article, we briefly review decades of research on social-cognitive development of children with hearing loss acquired in infancy, discuss how access to language-mediated communication contributes to the emergence and expression of understanding other minds and highlight some implications for effective interventions.

## INTRODUCTION

Researchers have long been interested in studying the relationship between environmental factors and individual development, particularly in the context of sensory input loss or reduction. Specifically, the experiences of individuals with hearing loss provide a natural context to investigate how reduced auditory input shapes learning, social interaction, and neuro-biological development. Evidence from children with hearing loss helps to uncover adaptive strategies employed in the absence of typical sensory cues, which shed light on the plasticity of the human brain. Furthermore, investigating language acquisition, social cognition, and emotional development in children with hearing loss reveals how sensory experiences influence cognitive and socioemotional functioning.

Additionally, insights derived from studying children with hearing loss have practical implications for designing interventions and educational approaches tailored to their unique needs. By understanding the specific challenges faced by this population, researchers and educators can develop targeted strategies to support optimal development and mitigate potential difficulties associated with auditory deprivation.

## VARIETIES OF AUDITORY EXPERIENCES IN DEAF INDIVIDUALS

Hearing loss, congenital or acquired in early infancy, can significantly influence language acquisition and cognitive development (Lederberg et al., 2013; Moeller & Tomblin, 2015). Unilateral and bilateral hearing loss present different dimensions of auditory experiences, with unilateral hearing loss leading to challenges in sound localization and speech understanding in noisy environments, and bilateral hearing loss profoundly impacting overall auditory perception, hindering communication and social interactions (Laugen et al., 2017). The degrees of hearing loss, ranging from mild to profound, further contribute to the complexity of access to auditory stimuli, directly influencing the individual’s ability to perceive and process auditory information (Moeller, 2007). Various types of hearing aids aim to mitigate the loss of access to auditory stimuli. While traditional hearing aids amplify sound for those with residual hearing, cochlear implants directly stimulate the auditory nerve, bypassing damaged hair cells in the inner ear (Bat-Chava et al., 2005). The utilization of these devices depends on factors such as the individual’s type and degree of hearing loss, as well as lifestyle considerations (Rauschecker & Shannon, 2002).

## DEAF EDUCATION AND DIVERSE SCHOOLING MODELS

The historical landscape of deaf education has witnessed an evolution in methodologies, reflecting changing societal perceptions and advancing insights into the linguistic needs of deaf individuals. Early approaches leaned towards oralism, emphasizing spoken language acquisition and speech training as the primary educational goals (Gulliver, 2005; Moores & Miller, 2009), but sign language was gradually recognized as a legitimate linguistic medium for deaf individuals. This acknowledgment prompted the emergence of bilingual education models, recognizing the significance of fostering proficiency in both a sign language and the spoken language of the surrounding hearing community.

Further developments in educational approaches include the use of Sign Supported education in which sign language alongside spoken language is used, providing a multi-sensory approach that supports language comprehension and expression. This method aims to bridge the gap between oralism and sign language, acknowledging the benefits of both communication modes (Gulliver, 2005; Moores & Miller, 2009).

The choice of language in deaf education plays a pivotal role in children’s social and cognitive development. Language is not merely a tool for communication, but a fundamental aspect of identity and cultural expression. For deaf children, the selection of an appropriate language model, whether it be sign language, spoken language, or a combination, significantly influences their ability to engage with their peers, educators, and the broader community. The historical trajectory of deaf education, marked by shifts between oralism, sign language recognition, and bilingual approaches, underscores the ongoing process to tailor educational methodologies to the diverse linguistic needs of deaf individuals.

## FROM HEARING AIDS TO COCHLEAR IMPLANTS

The history of hearing aids has significantly impacted the landscape of deaf education, language accessibility, and the deaf culture (Moores, 2012). Early attempts at amplifying sound for individuals with hearing loss date back to the 17th century, with rudimentary ear trumpets and speaking tubes. However, it was in the 20th century that transformative breakthroughs began to shape the field.

The introduction of electronic hearing aids in the mid-20th century marked a turning point. These devices utilized microphones and amplifiers to enhance auditory signals, providing individuals with hearing loss a means to access a broader range of sounds (Valentinuzzi, 2020). While these early devices represented a considerable leap forward, they had limitations in addressing severe or profound hearing loss—until the advent of cochlear implants. First developed in the late 20th century, cochlear implants are surgically implanted devices that directly stimulate the auditory nerve, bypassing damaged hair cells in the inner ear (Clark, 2003).

The introduction of cochlear implants has had multifaceted effects on deaf education, language access, and the deaf culture. In education, these have provided new opportunities for students with hearing loss, allowing them to engage more fully in conventional classroom settings. The emphasis on spoken language development with cochlear implants has sparked discussions about language choices within the deaf community, particularly regarding the use of sign language versus oral communication (Marschark & Spencer, 2016). Questions about the impact on deaf culture, identity, and the perception of deafness as a variation rather than a deficiency have been central to these debates (Moores, 2012). Technological advancements have not only altered the educational landscape for deaf individuals but have also prompted critical reflections on the complex interplay between technology, language access, and the preservation of deaf culture.

## RESEARCH ON PERFORMANCE ON ELICITED-RESPONSE (‘TRADITIONAL’) FALSE-BELIEF TASKS

Traditional false belief tasks have been a cornerstone of research in social cognition and theory of mind for decades. These tasks are designed to assess the ability to attribute true and false beliefs to themselves and others by means of explicit test questions. False belief tasks test whether individuals can recognize that others can hold beliefs that are incorrect or outdated, and that these beliefs can influence their behavior. For the purpose of this paper, we will focus on two categories of traditional false belief tasks. The first category includes first-order false belief tasks, which involve understanding that another person can hold a belief that is different from reality. Examples of these tasks include scenarios involving *unexpected contents*, *unexpected transfer*, and *changed appearance* tasks. The second category comprises advanced false belief tasks, which require a more sophisticated understanding of mental states and social interactions. These tasks include *second-order false belief*, *faux pas*, *strange stories*, *double bluff*, and *sarcasm* (each described in detail below).

Research on the development of theory of mind in children with hearing loss suggests a delay in this ability, which is generally attributed to restricted opportunities for learning through conversations about mental states. In their seminal study, Peterson and Siegal (1995) focused on 26 severely and profoundly prelingually deaf Australian children (*M* = 10 years 7 months) of which 24 had hearing parents (see Table 1). They were administered two versions of the most widely used standard false belief task, often referred to as the *Sally-Anne* or *unexpected transfer* task (Baron-Cohen et al., 1985). In this scenario, a character (e.g., Sally) places an object in one location and leaves the scene. Another character (e.g., Anne) moves the object to a different location while Sally is away. Sally returns, and the child is asked where Sally will look for the object. To pass this task, children must understand that Sally holds a false belief about the object’s location and will look in the original spot, not where the object actually is. Two control questions are used to ensure the child’s understanding of the scenario: *Where is the object really?* and *Where was the object in the beginning?*.

**Table 1.** Studies included in the review

**Reference**	***N***	**Age Range**	**Mean age**	**Degree of hearing loss**	**Hearing aid**	**Parent language use**	**How ToM tests were administered**	**Task(s) used**	**Main findings**
Figueroa et al. (2020)	CI: 36	12–16	14;0	Prelingually deaf	CI_age_ = 2;3	N/A	SpL	UT less verbal	TH > CI
	TH: 54	12–16	13;6						TH = CI_early_; TH > CI_late_
									CI_bilateral_ > CI_unilateral_; CI_bilateral_ = TH
Marschark et al. (2019)	CI: 46	College students	N/A	N/A	CI_age_ = 6;7	NS: 9	All groups in SL or SpL or both	Second order FB, sarcasm, dubble bluff; self-ratings of SL and SpL; Non-verbal IQ Woodcock-Johnson III	TH > CI or non-CI
	No CI: 48				HA: 43	LS: 81			
	TH :41				No aid: 11				
Meristo and Strid (2020)	NS: 9	2;2–6;2	4;3	>80 dB	No aid	SL	Non-verbally, eye tracking	UT non-verbal	NS > LS
	LS: 13	1;5–8;5	4;9	>80 dB	CI	SpL			NS = TH
	TH: 24					SpL			NS and TH above chance
									LS at chance
Meristo et al. (2007)	NS: 24	7;4–16;1	12;3	Prelingually deaf	N/A	SL	SL	UT, UC, second order FB, faux pas, strange stories (+ emotion understanding); RPM	Deaf children attended either a bilingual or an oralist school.
	LS: 37	8;0–15;11	12;3			SpL	SL		TH > LS Estonian bilingual
	TH: 26	6;1–15;4	10;9			SpL	SpL		TH > LS Swedish bilingual
									TH > NS Estonian oralist
									NS > LS Estonian bilingual
									NS > LS Swedish bilingual
									NS > NS Estonian oralist
									TH = NS Estonian bilingual
Meristo et al. (2012)	LS: 10	1;5–2;2	1;11	Moderate to profound	5CI_age_: 14 m	SpL	Non-verbally, eye tracking	UT	TH > LS in false belief trial
	TH: 10	1;7–2;4	1;11		5HA_age_: 12 m				TH = LS in true belief trial
Meristo et al. (2016)	CI: 8	3;11–8;5	6;5	Profound	CI_age_: 1;9	SpL	Non-verbally, eye tracking + verbally with toys in SpL	UT non-verbal	TH > CI or HA (non-verbal)
	HA: 7	4;0–7;11	5;8	45–65 dB	HA_age_: 1;10	SpL		UT × 3 verbal	CI and HA at chance (non-verbal)
	TH: 15	4;1–8;11	5;11			SpL		PPVT	TH above chance (non-verbal)
								Raven	TH = CI and TH > HA (verbal)
Morgan and Kegl (2006)	LS: 22	7;0–39;0	(1) 13;9	Severely to profoundly deaf	N/A	SpL	SL	UC drawings	UC: Early learners > late learners
			(2) 24;1					Narrative task	
								RPM	
								Sign language – teacher ratings	
Netten et al. (2017)	HL: 44	3;4–5;10	4;9	35–70 dB; age at detection 0;0 to 4;6	Bilateral HA-s all except one; Mean age at HA 2;2	N/A	89% spoken language, 11% sign-supported Dutch	UT, picture stimuli; Language skills – medical records and parent reports	TH > HL
	TH: 101	3;4–5;10	4;9						
Peters et al. (2021)	CI: 25	3;0–6;4	4;9	N/A	CI_age_ = 12 m	SpL		UT & UC	TH = CI (UC and UT)
	TH: 25	3;6–5;11	4;8			SpL		ToM Scale	TH = CI (ToM Scale)
								Language scales	
Peterson (2004)	CI: 13	4;2–11;2	8;0	>91 dB	CI_age_ = 2–5 y	SpL		2× UT & 2× UC	TH > CI, HA or ASD
	HA: 13	5;0–12;1	7;6	N/A		SpL		Verbal IQ; PPVT	CI = HA
	ASD: 9	5;3–12;6	8;6					Non-verbal IQ: the Goodenough-Harris	CI + HA = ASD
	TH: 17	4;1–5;8	4;10						
Peterson and Siegal (1995)	LS: 26	8;1–13;0	10;7	Severely and profoundly prelingually deaf		SpL: 24	Total communication, toy play	2× UT; the Goodenough-Harris and Raven for non-verbal IQ + RPM	LS < Down syndrome
						SL: 2			LS = ASD
Peterson and Siegal (1999)	LS: 34	5;6–12;8	9;4	Severe to profound		SpL	Each child’s prefered language for LS and NS	UT; UC; CA; the Goodenough-Harris for non-verbal IQ; teacher ratings of language for deaf. PPVT for TH	NS = OD = TH
	NS: 11	6;6–12;8	10;3	Severe to profound		SL			NS or OD or TH > LS
	OD: 14	6;10–13;2	9;2	Moderate to severe		SpL	SpL		LS = ASD
	ASD: 22	6;2–13;9	9;6						
	TH: 21	3;11–5;4	4;6						
Pluta et al. (2021)	CI: 45	3;3–8;3	5;5	>90 dB	CI_age_ = 13 m	SpL		UT less verbal	TH > CI_younger_
	TH: 94	3;3–7;3	4;6	prelingual					TH = CI_older_
Smogorzewska and Osterhaus (2021)	HL: 270	Longitudinal study, ages 7;6 to 9;6	7;6	Moderate to severe	All HL children used CI or HA	N/A	Written test battery	Second-order FB; Faux Pas	TD > DHH > MID
	TH: 250		7;6					Polish Language test for grammar, vocabulary, reading and writing	
	MID: 210		8;1						
Sundqvist et al. (2014)	CI_early_: 8	4;3–9;3	6;7	All CI	CI_age_ = 1;6	SpL	SpL	UT	TH > CI_early_ or CI_late_
	CI_late_: 8	4;5–9;6	7;1	M = 88 dB	CI_age_ = 3;4	SpL	SpL	PPVT	CI_early_ = CI_late_
	TH: 18	6;0–8;0	6;7			SpL	SpL	RPM	

This study is missing in the table.

CA = changed appearance; UT = unexpected transfer; UC = unexpected contents; PPVT = Peabody Picture Vocabulary Test; RPM = Raven’s Progressive Matrices; LS = late signers (i.e., deaf children from hearing families); NS = native signers (i.e., deaf children from deaf families); OD = oral deaf children (i.e., children growing up with spoken language); TH = typically hearing children; ASD = autism spectrum disorder; HL = children with hearing loss; MID = mild intellectual disability; CI = cochlear implants, or children with cochlear implants; HA = conventional hearing aids, or children with conventional hearing aids; CI_age_ = age of first cochlear implantation; HA_age_ = age of initial hearing aid use; SL = sign language; SpL = spoken language.

Ages are provided in years and months (3;4 should be interpreted as 3 years and 4 months).

Degrees of hearing loss according to American Speech-Language-Hearing Association (mild 26 to 40 dB; moderate 41 to 55 dB; moderately severe 56 to 70 dB; severe 71 to 90 dB; profound 91+).

In Peterson and Siegal (1995) this test was administered using Total Communication—a combination of signed English, Auslan, and speech with visible lip movements, translated by a professional interpreter, with the help of toys. Participants were screened for nonverbal IQ using the Goodenough-Harris Drawing Test (Harris, 1963) and they were within the typical developmental range. Overall, 35% of deaf children passed both versions of the task. Their performance was lower than children with Downs syndrome, and similar to children with ASD (Baron-Cohen et al., 1985). These results supported a conversational account of theory of mind development, with a lack of early social interactions suggested as a probable factor in the delayed comprehension of mental states. A wealth of research replicated these original discoveries (for a review see Peterson, 2009) and investigated additional questions. One of them was prompted by the observation that the two children in Peterson and Siegal’s (1995) study who were raised by deaf signing parents performed well in the false belief tests.

In their follow-up study, Peterson and Siegal (1999) divided 59 deaf children into three groups based on their communication modality. The first group consisted of profoundly deaf signers from hearing families, referred to as late signers (*N* = 34; *M*: 9 years 4 months). The second group included deaf native signing children who had at least one other deaf signing member in their home, known as native signers (*N* = 11; *M*: 10 years 3 months). The third group comprised children with moderate to severe hearing loss from hearing families, without any immediate family member using sign language, referred to as oral deaf children (*N* = 14; *M*: 9 years 2 months). Additionally, the study included autistic children (*N* = 22; *M*: 9 years 6 months) and typically hearing peers (*N* = 21; *M*: 4 years 6 months) as control groups. The tasks used in the study included the *unexpected transfer* task (Baron-Cohen et al., 1985), the *unexpected content* task (Perner et al., 1989), and the *changed appearance* task (Leekam & Perner, 1991). The *unexpected content* task (Perner et al., 1989), also known as the classic Smarties task, involves presenting a familiar sweets container that unexpectedly contains pencils instead of sweets. Participants are then asked to predict what a naive peer would think the container holds upon seeing it closed for the first time. Two control questions are used to ensure the child’s understanding of the scenario: *What is actually inside the container?* and *What did you think was inside the container before we opened it?*. The *changed appearance* task involves a scenario with a mother doll and a girl doll (Leekam & Perner, 1991). The mother comments on the girl’s yellow dress and leaves to find matching ribbons. While the mother is gone, the girl changes dresses. Children are then asked which ribbon the mother will bring (test question), what color the girl’s dress was when the mother left (memory control), and what color her dress is now (reality control).

In order to pass each task, children had to correctly answer both test and the following control questions. Additionally, teachers rated the deaf children’s expressive language skills, comprehension, and vocabulary size both in sign language and spoken language in scales of 1 (below average, inadequate for effective communication) to 5 (highly competent). Each child was assigned a verbal ability score derived from the three scales from their stronger modality. The hearing children’s language ability was measured with a vocabulary test (Dunn & Dunn, 1981).

The study found a significant difference in the number of false-belief tasks passed among various groups of children. There was no reliable difference between the performances of late signers (*M* = 1.44) and those of autistic children (*M* = 1.59). However, both groups performed worse than native signers (*M* = 2.73), oral deaf children (*M* = 2.21), and typically developing hearing preschoolers (*M* = 2.52), who did not show significant differences among themselves. This performance pattern was consistent across individual tasks, and the three deaf groups had no significant differences in their average verbal ability scores, age, or non-verbal IQ. The authors suggest that deaf children raised by deaf parents can have fluent conversations in sign language about referents that are not physically present, such as mental states, similar to hearing children with hearing parents. Subsequent research has confirmed these findings in relation to deaf children who have been exposed to skilled signers at home from birth and later tested on false belief tasks (Courtin, 2000; Schick et al., 2007).

In addition to studying profoundly and severely deaf children, researchers have also investigated children with moderate hearing loss. In a study conducted by Netten et al. (2017), an *unexpected transfer* false belief task supported by a drawing (Baron-Cohen et al., 1985) (as well as additional tasks about intention and desire understanding) was administered to a group of 44 children with moderate hearing loss (ranging from 35 to 70 dBs in the better ear) between the ages of 3 years 4 months to 5 years 10 months (*M* = 4 years 9 months). All but one child had conventional bilateral hearing aids (one child used a bone conduction device). Additionally, seven of the children had parents with hearing loss themselves. Although all children understood spoken language, five of them (11.4%) preferred to use sign-supported Dutch during the testing. The control group included 101 children aged 3 years 4 months to 5 years 10 months (*M* = 4 years 9 months). The expressive language scores for children with hearing loss were obtained from their medical records, and the study therefore did not include comparable language data for the hearing control group. Children with hearing loss scored within the normal range on these tests. However, on parent-reported measures using the Dutch versions of the Child Development Inventory (Ireton & Glascoe, 1995) children with hearing loss scored lower than hearing controls on both expressive language and language comprehension. The study did not provide any information about the non-verbal intelligence or cognitive abilities, however no child had developmental disabilities.

Children with hearing loss scored significantly lower on the false belief task (*M* = 0.44) compared to hearing peers (*M* = 0.63, max = 1.0). False belief understanding increased with age in both groups. The overall model was statistically significant, indicating that together, group and age were significant predictors of false belief understanding. False belief understanding showed a significant positive correlation with both language comprehension and expressive language as reported by parents for both groups together after controlling for age. These findings underscore the potential impact of even moderate hearing loss on social-cognitive development to support perspective-taking skills in this population. However, the study does not indicate whether any children failed the control questions or were excluded from analysis, nor does it report if either group’s performance on the false belief task was significantly above chance level, which limits the interpretation of the results.

Theory of mind skills have also been examined in older children with hearing loss and adults. Meristo et al. (2007) investigated the performance of 61 deaf children in Estonia and Sweden, including 24 native signers (i.e., children who were born to deaf parents and learned sign language from birth) and 37 late signers (i.e., children who were born to hearing parents and learned sign language later in life). The native signers attended either bilingual schools (*N* = 11; *M* = 12 years, 10 months) or oralist schools (*N* = 13; *M* = 11 years, 9 months), while the late signers attended bilingual schools in Estonia (*N* = 16; *M* = 12 years, 1 month) or Sweden (*N* = 21; *M* = 12 years, 4 months). The bilingual schools used both a sign language and a spoken language as mediums for instruction, whereas the oralist schools focused on spoken language instruction, often relying on lipreading. All the deaf children primarily used either Estonian Sign Language or Swedish Sign Language as their main and preferred means of communication. Their teachers had assessed them as fluent in these sign languages, despite the lack of a formal test of language proficiency. All children were prelingually deaf and were tested in their own sign language.

The test materials included *unexpected transfer* and *unexpected content* false belief tests, as well as more advanced theory of mind tests such as two *second-order false belief* (*faux pas* and *strange story*; Baron-Cohen et al., 1999; Happé, 1994; Hughes et al., 2000; Sullivan et al., 1994), and three additional emotion recognition tasks (Harris et al., 1989; Howlin et al., 1999).

The *second-order false belief task* involves a grandfather giving his grandchildren chocolate and asking them to put it away until their mother says they can have some (Hughes et al., 2000). The children hide it in the fridge and go outside. Later, the boy moves the chocolate to his bag, unaware that his sister sees him do this through the window. When their mother later says they can have the chocolate, children are asked: *Where does the boy think the girl will look for the chocolate?* To pass, children must understand that the boy doesn’t know his sister saw him hide the chocolate. This tests their ability to reason about a character’s mistaken belief about another character’s knowledge—a more advanced theory of mind skill compared to simpler first-order false belief tasks. *The faux pas task* involved presenting children with a story about a social situation where someone unintentionally says something inappropriate or hurtful, requiring understanding of multiple characters’ mental states (Baron-Cohen et al., 1999). In this study, the story described a character who made critical remarks about a birthday present (a toy airplane) without realizing they were speaking to the person who had given them the gift. Children were asked if someone said something improper in the story, and if so, what it was. In *the strange story task* (Happé, 1994), children are presented with a narrative where a character’s actions are misinterpreted due to a false belief. For example, an old woman mistakenly believes a man approaching her intends to harm her, when in fact he just wants to ask for the time. Children are asked to explain why the woman reacted as she did, requiring them to understand the character’s false belief and its consequences.

The theory of mind score was calculated by summing the correct responses across all eight tasks resulting in a total score ranging from 0 to 8. There were no significant differences in nonverbal mental age (Raven, 1962) or chronological age between the five groups. The results of the theory of mind total score showed that hearing children (*M* = 6.56, 82%) significantly outperformed late-signing Estonian bilingual deaf children (*M* = 4.44, 56%), late-signing Swedish bilingual children (*M* = 4.98, 62%), and native-signing Estonian oralist children (*M* = 4.46, 56%). However, there was no significant difference between hearing children and bilingually instructed native signers (*M* = 6.59, 82%). The bilingually instructed native signers significantly outperformed bilingually instructed Estonian late signers, bilingually instructed Swedish late signers, and oralist-instructed native signers. The results were reanalyzed using ANCOVA with chronological age and nonverbal mental age as covariates, which revealed that the group differences in theory of mind performance remained significant even when these factors were held constant. The authors suggest that early and continuous access to a sign language, particularly in a bilingual school environment, plays a crucial role in the development of theory of mind reasoning in deaf children.

These findings about theory of mind delays in older deaf children have been confirmed recently in a longitudinal study with 270 deaf children followed over a two-year period from age 7 years 6 months to 9 years 6 months (Smogorzewska & Osterhaus, 2021). The study examined the development of advanced theory of mind, compared to typically developing children (*n* = 250) and children with mild intellectual disability (*n* = 210). The deaf group comprised 108 children with profound hearing loss (>80 dB), 110 with severe loss (61–80 dB), and 52 with moderate loss (41–60 dB). All deaf children used sign language to support spoken language, used at least some spoken language to communicate, had no developmental disabilities, and had hearing aids and/or cochlear implants (no detailed numbers for each type of device provided). The study employed a second-order false belief task (Hughes et al., 2000) and ten faux pas recognition tasks (Baron-Cohen et al., 1999). To earn a score, children were required to answer all test and control questions correctly. The faux pas stories used at different time points were mostly distinct, with only two stories repeated longitudinally (maximum score = 2). Language abilities were assessed at the initial time point using a non-standardized Polish language task, which consisted of 17 tasks that evaluated children’s grammar and vocabulary skills, as well as their ability to recognize letters, write, and read.

The descriptive statistics showed that typical children performed higher than both other groups on second-order false belief and faux pas recognition tasks at all time points. At age 7.5, 57% of typical children passed the second-order false belief task, compared to 27% of children with intellectual disability and 38% of deaf children. By age 9.5, these percentages increased to 81%, 45%, and 63% respectively. For faux pas recognition, at age 7.5, typical children scored 0.82 out of 2, compared to 0.41 for children with intellectual disabilities and 0.61 for deaf children. By age 9.5, these scores increased to 1.41 for typical children, 0.96 for children with intellectual disabilities, and 1.19 for deaf children.

The piecewise latent trajectory model revealed that the intercepts differed significantly among the groups, with typical children having the highest intercept and children with intellectual disabilities having the lowest. There was a significant difference in intercepts between typical hearing children and deaf children. The slopes were significant in all three groups, but the differences in slopes between the groups were not significant. That is, despite the initial differences in faux pas performance, the speed of development did not differ across the groups. This suggests that children with intellectual disabilities and deaf children do not catch up with their typically developing hearing peers during the time span of the study. There were also significant correlations between theory of mind measures and language abilities, particularly in the group of deaf children.

This two-year longitudinal study found significant differences in advanced theory of mind development between typically developing children, children with mild intellectual disabilities, and deaf or hard of hearing children aged 7.5 to 9.5 years. A key limitation is the lack of control for other cognitive abilities beyond language, such as executive function, which are known to relate to theory of mind development. Also, the tasks required understanding of very complex written language, which raises questions about whether they truly measured theory of mind or were more reflective of language comprehension abilities in this group. Encouragingly, by age 9.5, deaf children performed at a high level on these complex tasks, despite still lagging behind their typically hearing peers. This indicates meaningful development of these critical social-cognitive skills in deaf children during middle childhood, even if delays persist.

Marschark et al. (2019) examined theory of mind abilities in deaf and hearing college students. Their study involved 94 deaf and 41 hearing students. Of the deaf participants, 46 were active cochlear implant users, with a mean age of first implantation at 6 years 7 months. Regarding sign language, 90 deaf participants reported knowing it, with 9 being native signers and 81 learning it between ages 5 and 22 years (*M* = 6 years 11 months). The mean age of sign language acquisition was 7 years 1 month for implant users and 5 years 5 months for non-users. Fifty-eight deaf participants (27 implant users, 31 non-users) reported sign language as their best form of communication, while 35 (19 implant users, 16 non-users) preferred spoken language. Four deaf students reported knowing no sign language.

The theory of mind tasks included three advanced tasks. First, four stories of understanding *sarcasm*, which tested participants’ ability to recognize sarcastic statements and explain why the character used sarcasm. For example, participants had to explain why a character sarcastically said it was a lovely day for a picnic when it was actually raining (O’Reilly et al., 2014). Second, two *second-order false belief stories*, which required participants to understand recursive mental states—what one character thinks another character believes (Coull et al., 2006; O’Reilly et al., 2014). Third, two *double bluff* stories, which required participants to understand deception through telling the truth. For example, participants had to explain why a character who usually lies chose to tell the truth in order to deceive (Happé, 1994). Participants’ performance on stories was evaluated using a control question for factual comprehension followed by a test question for theory of mind understanding. Analysis showed that hearing participants performed significantly better than both groups of deaf participants on sarcasm and second-order false belief on control questions, but there was no significant difference on double bluff test. The participants’ language skills were measured through self-assessments using a 5-point scale for both spoken and sign languages. Participants also completed tests of visual-spatial processing and sequential processing to assess potential cognitive factors related to theory of mind performance: the Woodcock-Johnson III Spatial Relations task (Woodcock et al., 2001), and the General Ability Measure for Adults Sequences subtest (Naglieri & Bardos, 1997).

The study found that hearing participants outperformed both cochlear implant users and deaf nonusers on all tasks, while there were no significant differences in performance between the two deaf groups on any of the tasks. For deaf participants, self-rated spoken language ability was a significant predictor of sarcasm understanding, while Sequences scores predicted second-order false belief understanding. No significant predictors were found for double bluff understanding. Notably, neither implant use nor sign language ability was a significant predictor of theory of mind performance in deaf participants, nor was the length of implant use or the age of sign language acquisition. The small group of native signers did not differ significantly from other deaf participants in theory of mind performance. According to the authors, despite access to spoken language through cochlear implants or early exposure to sign language, deaf young adults exhibit persistent delays in advanced theory of mind understanding relative to hearing peers. However, there were notable group differences in responses to control questions, and since children who did not answer correctly to these questions were included in the final analysis, it remains unclear whether any group differences would persist in the test questions. Another critique of the study is that the testing format, likely written and sometimes conducted in small groups, may have biased the results by relying on participants’ written language skills, which were not directly assessed. This is particularly relevant since spoken language skills (and not sign language skills) were found to predict performance on the sarcasm task, suggesting that language proficiency could be a significant factor in the outcomes.

Finally, Morgan and Kegl (2006) investigated the relationship between language development and theory of mind in 22 deaf Nicaraguan children and adults who learned Nicaraguan Sign Language either before age 10 (early learners, *n* = 11, *M* = 13 years 11 months) or after age 12 years (late learners, *n* = 11, *M* = 12 years 1 month) and who showed no deficits in non-verbal intelligence. Teacher judgments indicated that participants’ competence in Nicaraguan Sign Language grammar was appropriate for their age. Additionally, both groups of signers demonstrated comparable linguistic complexity in their narratives, despite differences in mental state propositions mentioned.

The study used a false belief task with *thought bubbles* in cartoon scenarios, which is less verbally demanding than other traditional tasks (Woolfe et al., 2002). In this task the participants are shown a series of scenarios where a character believes something that is not true, and they must identify what the character is thinking about by choosing from a set of pictures. For example, in one scenario, a boy is fishing and thinks he has caught a fish, but it is actually an old boot. The participant must point to the picture that shows what the boy is thinking about, which in this case would be a fish. There were two false belief and two true belief trials; all participants answered correctly in both true belief trials. Participants also completed a narrative task, retelling a 1.5-minute non-verbal cartoon to a fluent adult signer, with their signed narratives transcribed and coded for references to mental states.

Results showed that early learners significantly outperformed late learners on the false belief task, with 10 out of 11 early learners passing compared to only 4 out of 11 late learners (*M* = 1.81 and *M* = 0.73). However, both groups demonstrated some theory of mind abilities in the narrative task, with even late learners able to talk about different types of mental states. However, late learners often omitted *lack of knowledge* and *deception* mental states, which are related to false belief.

The authors conclude that early language exposure is crucial for developing false belief understanding, but late learners can still develop other important theory of mind abilities. Although the researchers acknowledge that their study is limited by the lack of standardized measures of Nicaraguan Sign Language fluency and the potential for linguistic rather than conceptual reasons for task performance, their findings suggest a strong relationship between language exposure and theory of mind development.

## THE IMPACT OF COCHLEAR IMPLANTS ON THEORY OF MIND DEVELOPMENT

Studies on the impact of cochlear implants on the development of theory of mind in children with hearing loss have reported inconsistent results. Some studies suggest that children with implants may still experience delays in theory of mind development, similar to their peers with hearing aids. Peterson (2004) examined theory of mind development in oral deaf children with cochlear implants or hearing aids, compared to children with autism and typically developing preschoolers. Their sample included 13 deaf children with cochlear implants (*M* = 8 years 0 months), 13 deaf children with hearing aids (*M* = 7 years 6 months), 9 children with autism (*M* = 8 years 6 months), and 17 hearing preschoolers (*M* = 4 years 10 months). The ages at implantation for the implant group ranged from 2 to 5 years. None of the deaf children had older signing deaf family members, suggesting they likely used spoken language at home with their parents. For the deaf children from mainstream schools, the false belief tasks were administered using oral speech that was fully enunciated with visible lip movements. For those from Total Communication classrooms, tasks were administered using simultaneous speech and manual translation into grammatically complete Signed English. Participants completed a battery of two standard false belief tests: the *unexpected location* false belief task (2 trials, each including one test question followed by two test questions) (Baron-Cohen et al., 1985) and the *unexpected content* false belief task (2 trials, each including two control questions followed by two test questions) (Gopnik & Slaughter, 1991). For both tasks, children who passed the control questions could earn a score of 1 for each correctly answered test question. Results showed that deaf children with cochlear implants performed similarly to deaf children with hearing aids. Both groups of deaf children combined scored comparably to the autism group and each deaf group scored significantly below hearing preschoolers, despite being older. Only 8% of cochlear implant users and 38% of hearing aid users passed all theory of mind tasks, compared to 71% of hearing preschoolers. Verbal mental age was significantly correlated with theory of mind performance for the sample as a whole. Multiple regression analyses revealed that verbal ability predicted theory of mind scores even after controlling for age and disability diagnosis.

Similarly, Sundqvist et al. (2014) conducted a study on theory of mind development in children with cochlear implants. The sample included 16 children with cochlear implants (*M* = 6 years 6 months) and 18 typically developing children (*M* = 6 years 7 months). The implanted group was divided into early (implanted before 2 years 3 months, *M* = 1 year 6 months) and late (implanted after 2 years 3 months, *M* = 3 years 5 months) subgroups. The two groups did not differ in terms of the length of implant use. The children had a spoken Swedish-speaking home environment. Twelve of the children attended mainstream schools in their neighborhood, three attended classes for children with hearing impairments, and one child attended a state school for the deaf. The groups did not differ significantly on nonverbal mental age (measured with Colored Progressive Matrices; Raven, 1962) or receptive vocabulary (Peabody Picture Vocabulary Test (Dunn & Dunn, 1981) in spoken Swedish). Participants completed an *unexpected transfer* false belief task consisting of three test questions and were awarded a maximum of 3 points if the three control questions were answered correctly as well.

An ANOVA revealed a significant main effect for the unexpected transfer task. Post-hoc comparisons indicated that both implant groups differed significantly from the comparison group (early: *M* = 41.6%, late: *M* = 20.9% vs. controls: *M* = 96.2% correct), but there was no significant difference between the early and late implant groups (although the early implant group performed similarly to typically developing children on emotion understanding, while the late implant group scored significantly lower). There was a positive correlation between the nonverbal intelligence and the unexpected transfer task, but no correlation between the vocabulary and unexpected transfer. The findings suggest that both early and late implanted groups show delays in theory of mind development compared to typically developing peers. It is important to note some limitations of this study, including the small sample size. Additionally, even the *early* implanted group started to use implants relatively late (*M* = 1 year 6 months) compared to current Swedish standards, where implantation often occurs before 12 months of age. Finally, since children who failed the control questions were not excluded but rather scored zero, it’s possible that some lower scores may reflect difficulties understanding task instructions rather than true deficits in theory of mind abilities.

However, other research has indicated that the use of cochlear implants from a relatively early age may facilitate theory of mind development. One study by Meristo et al. (2016) investigated the development of false belief in deaf and hearing children aged 4-8 years. The participants included 15 typically hearing Swedish children (*M* = 5 years and 11 months); 8 Estonian children with cochlear implants (*M* = 6 years 5 month) who were implanted at a mean age of 1 year and 9 months (range: 1 year 4 months–2 years 9 months) and had used their implants for a mean of 4 years 8 months; and 7 Estonian children with hearing levels in the moderately deaf range using hearing aids (*M* = 5 years 8 months). Children were tested on three versions of the *unexpected transfer* task using spoken language. To score a point, they had to answer both the test and following control questions correctly. However, children who failed to answer the control questions correctly were still included in the analysis.

The study found a significant difference among the groups with typically hearing children (*M* = 2.4) significantly outperforming the hearing aid children (*M* = 0.4). However, there was no significant difference between the hearing children and the cochlear implant children, (*M* = 1.6). Language development, measured by the Peabody Picture Vocabulary Test (Dunn & Dunn, 1981), was significantly correlated with the unexpected transfer task for the entire sample. Hearing children outperformed the deaf groups, but there were no statistically significant differences among the groups in non-verbal IQ (Raven, 1962), and no differences between cochlear implant and hearing aid groups in verbal IQ (the Peabody Picture Vocabulary Test).

The authors suggest that language quality and parents’ interactive skills play a significant role in the development of theory of mind in both hearing and deaf children. As with many studies including children with hearing loss, the relatively small number of participants might have affected the robustness and generalizability of the findings. Yet, upon closer examination, the results from Sundqvist et al. (2014) and Meristo et al. (2016) may not be as contradictory as they initially appear, with Sundqvist’s early implant group showing a 46% passing rate and Meristo’s cochlear group scoring 1.6 out of 3 points (equivalent to 53%), suggesting some level of theory of mind development in both studies despite the differences in methodology and reporting.

In another recent study, Peters et al. (2021) investigated theory of mind acquisition in young children who are deaf with hearing parents and implanted early, compared to age-matched children with typical hearing. The participants included 25 children with cochlear implants aged 3 years 0 months to 6 years 4 months (*M* = 4 years 9 months) and 25 children with typical hearing aged 3 years 6 months to 5 years 11 months (*M* = 4 years 8 months). The children with implants started to use their first implant between 6 and 18 months of age (*M* = 1 year 0 months), and had been using their implants for an average of 3 years 9 months at the time of testing. Theory of mind was evaluated using a modified version of the five-item scale developed by Wellman and Liu (2004), with an additional false belief task. The tasks included Diverse Desires, where children have to understand that different people can want different things; Diverse Beliefs, which tests children’s recognition that people can have different thoughts about the same situation; Knowledge Access, which assesses children’s understanding that seeing leads to knowing; and Real-Apparent Emotion, which evaluates children’s understanding that a person’s facial expression may not match their true feelings. All children with cochlear implants used spoken English as their primary mode of communication, and all tests were administered in spoken English.

Results showed no significant differences between the two groups on measures of expressive language or receptive language. The only significant difference was in hearing age, with deaf children having been exposed to spoken language for approximately 12 months less than their hearing counterparts. On the 6-item theory of mind scale, children who were deaf achieved a mean score of 3.80, while typically hearing children scored 3.96. This difference was not statistically significant. Two false belief tasks were included in the study: the *unexpected content* task and the *unexpected transfer* task. Results showed that 36% of children who were deaf passed the unexpected contents task, compared to 48% of children with typical hearing. For the unexpected transfer task, 60% of children who were deaf passed, while 68% of children with typical hearing were successful. For the children who were deaf, expressive and receptive language skills were significantly correlated with theory of mind performance, even after controlling for chronological age.

The authors suggest that children who get cochlear implants early and have good language skills are more likely to acquire theory of mind in a typical time frame. However, given the low percentages of children passing the unexpected contents false belief tasks in both groups, it is likely that neither group performed significantly above chance level. Additionally, the study’s scoring method, where children pass a test by answering a single question correctly without any control questions, may lead to an overestimation of their performance compared to previous studies. Most other studies require passing control questions to ensure understanding and memory. This difference in methodology could potentially inflate the success rates reported in the current study.

Pluta et al. (2021) investigated false belief understanding in 45 children with cochlear implants aged 3 years 3 months to 8 years 3 months (*M* = 5 years 5 months) who started to use their first implant between 6 months and wo years 3 months of age (*M* = 1 year 1 months), and 94 typically hearing children aged 3 years 3 months to 7 years 3 months (*M* = 4 years 6 months). Although no formal tests of general non-verbal cognitive abilities were included in the study, all children were screened by qualified professionals to ensure they had no intellectual disabilities or developmental delays. All deaf children had prelingual, sensorineural hearing loss and parents with typical hearing levels who did not use sign language to communicate.

Participants completed a modified computerized *unexpected transfer* false belief task. This task consisted of three conditions: false belief, true belief, and no belief. In each condition, participants watched short cartoon clips depicting a character observing a toy being placed in a box. In the false belief condition, the character leaves and is absent when the toy moves to another box. In the true belief condition, the character sees the toy move. The no belief condition used a geometric shape instead of a character. Each condition was presented four times, allowing participants to score 0–4 points per condition. The test question asked where the child thinks the toy is (false/true belief) or which box contains the toy (no belief). To reduce linguistic and executive demands, the scenarios were presented as cartoons without elaborate storylines, and responses were given by touching a box on a touchscreen. Children who did not answer at least two of the four control questions correctly were excluded from further analysis to control for language and memory demands of the task (i.e., answered at most one question correctly on the no belief condition). All children also completed a sentence comprehension test involving pointing to pictures matching spoken sentences of varying grammatical complexity.

Results showed that 4- and 5-year-old children with implants performed significantly worse on the false belief condition than their hearing peers (*M*_implant_ = 0.88; *M*_*hearing*_ = 2.09). However, this difference was not observed in the older age group (6–8 years), where children with implants performed similarly to hearing children (*M*_implant_ = 2.08; *M*_*hearing*_ = 3.00). No significant differences were found between groups in the true belief or no belief conditions for either age group. Sentence comprehension was significantly lower in children with implants compared to hearing peers in both younger (*M*_implant_ = 18.38, *M*_*hearing*_ = 25.11) and older age groups (*M*_implant_ = 25.39, *M*_*hearing*_ = 28.89). Age at implantation did not significantly correlate with false belief performance, but showed a negative relationship with sentence comprehension after controlling for age. Duration of implant use correlated positively with both false belief and sentence comprehension, though the false belief correlation became non-significant after controlling for age. Overall, the results suggest that children with cochlear implants eventually catch up on theory of mind development, but interventions to support theory of mind skills may be beneficial.

Finally, a study by Figueroa et al. (2020) examined theory of mind abilities in Spanish adolescents with cochlear implants aged 12–16 years compared to typically hearing peers. The sample included 36 adolescents with implants (*M* = 14 years and 0 months) and 54 hearing adolescents (*M* = 13 years and 6 months). The implant group was further divided into early-implanted (*N* = 13, implanted at a mean age of 1 year and 5 months) and late-implanted (*N* = 23, implanted at a mean age of 2 years and 9 months) subgroups. Additionally, the cochlear implant group was categorized into bilateral (*N* = 13, with bilateral implants or an implant and a hearing aid) and unilateral (*N* = 23, with unilateral implants) subgroups. Theory of mind was evaluated using an unexpected transfer false belief task adapted from Ryskin and Brown-Schmidt (2014). In this task, participants viewed two pictures and read a story about a character named Vicki who places a violin in a blue box before leaving the room. Her sister then moves the violin to a red box and rearranges the boxes. Participants had to indicate where Vicki would look for the violin upon returning by assigning percentage likelihoods to each colored box. The correct response was to assign the highest percentage to the blue box, indicating understanding of Vicki’s false belief. The score was calculated by subtracting the percentage assigned to the red box from the percentage assigned to the blue box, with higher scores indicating better understanding of the false belief. Results indicated that the typically hearing group outperformed both cochlear implant groups. The hearing group (*M* = 56) scored significantly higher than the late-implanted group (*M* = 35). However, there was no significant difference between the hearing group and the early-implanted group, (*M* = 49). Additionally, bilateral implant users (*M* = 45) performed better than unilateral users (*M* = 37). The performance of bilateral implant users was comparable to that of the typically hearing group, suggesting that early implantation and bilateral auditory input may enhance theory of mind abilities in adolescence.

In summary, research on the impact of cochlear implants on false belief development in children with hearing loss has yielded mixed results. Some studies have found that children with implants experience delays in theory of mind development compared to their typically hearing peers. However, other research suggests that early implantation may facilitate theory of mind development, with some implant users performing similarly to hearing children. Factors such as age at implantation, duration of implant use, and language skills appear to play crucial roles. The overall take-home message is that while children with implants may initially show delays in theory of mind development, many seem to catch up over time, especially those who start using implants early and develop strong language skills. Nevertheless, individual variability exists, and some children with implants may benefit from targeted interventions to support their theory of mind development.

## RESEARCH ON PERFORMANCE ON SPONTANEOUS-RESPONSE FALSE-BELIEF TASKS

Spontaneous-response false-belief tasks, which assess understanding relying on spontaneous behaviors such as looking patterns (Surian et al., 2007), emotional expressions (Scott, 2017), helping (Buttelmann et al., 2009) or pointing (Southgate et al., 2010), are easier for young children compared to elicited-response tasks, which require children to verbally answer questions about an agent’s false belief and arguably involve additional executive-function processes like inhibition and response selection. Research on the performance of children with hearing loss in spontaneous-response false-belief tasks is scarce and all comes from Meristo and colleagues (Meristo & Strid, 2020; Meristo et al., 2012, 2016, 2024). These studies have used anticipatory looking methods developed by Southgate et al. (2007) and by Surian and Geraci (2012), and followed the logic of traditional false belief tasks (Wimmer & Perner, 1983). Tasks, however, are totally non-verbal and children are looking at a screen while their eye movements are being recorded.

In Meristo et al. (2012) this measure consisted of an animated movie where a cat was chasing a mouse through a Y-shaped tunnel. In the false belief condition, the mouse first hid in one of the boxes located outside each of the exit points while the cat waited at the entrance of the tunnel. Next the cat moved off scene, and the mouse moved into the second box. Finally, the cat reappeared, entered the tunnel, and after 3 seconds left the tunnel through either one of the two exits. Children’s eye movements were measured from the moment the cat entered the tunnel until it exited. If children looked longer at the correct exit point (i.e., the one where the mouse was hiding when the cat saw it) as compared to the incorrect one, they can be assumed to have an understanding of false beliefs. This setup is particularly suitable for toddlers who have hearing loss, since it does not rely on children’s language and auditory abilities. While deaf children’s lower performance in traditional elicited response false belief tasks can be attributed to their difficulties in comprehending the test questions or formulating a verbal answer (Meristo et al., 2007; Peterson & Siegal, 1995), this interpretation can be ruled out in spontaneous-response tasks.

Meristo et al. (2012) administered this task to a group of ten infants with moderate to profound hearing loss (*M* = 1 year 11 months; range: 1 year 5 months to 2 years 2 months) and a control group of ten typically hearing children (*M* = 1 year 11 months; range: 1 years 7 months to 2 years 4 months). Among the children with hearing loss, half had been equipped with cochlear implants at a mean age of 14 months and had a mean duration of use of 7 months. The other half used traditional hearing aids, with an initial use at a mean age of 12 months and a mean duration of use of 14 months. All children had been born to hearing parents and communicated in spoken Swedish. None of them showed proficiency in sign language, and all were healthy, without known additional disabilities.

Results showed that hearing infants significantly outperformed infants with hearing loss in anticipating the search actions of the cat according to its false belief. In the false belief condition, hearing infants looked significantly longer at the correct than at the incorrect exit, while infants with hearing loss incorrectly anticipated that the cat would look for the mouse in its real location. In contrast, there was no significant difference between the two groups in the true belief condition. Additionally, 8 out of 10 infants in each group looked first at the correct location in the true belief condition, whereas in the false belief condition, all 10 hearing infants but none of the 10 hearing loss infants looked first at the correct location. The study concludes that hearing infants significantly outperform infants with hearing loss from hearing families in attributing false beliefs, highlighting the importance of early language exposure and conversational input for the development of theory of mind reasoning.

In a follow-up study, Meristo et al. (2016) investigated spontaneous and elicited false belief understanding in deaf and hearing children aged 4-8 years. Participants included 15 typically hearing Swedish children (*M* = 5 years 11 months), 8 Estonian children with cochlear implants (*M* = 6 years 5 months), and 7 Estonian children with conventional hearing aids (*M* = 5 years 8 months). The implanted children had started to use their first implants at a mean age of 1 year 9 months and had been using them for an average of 4 years 8 months. Five had bilateral and three had unilateral implants. The children with hearing aids started using them at a mean age of 1 year and 10 months and had been using them for an average of 5 years. All deaf children had hearing parents and no deaf relatives. The cochlear implant and hearing aid children were primarily exposed to spoken Estonian, though two preferred Estonian Sign Language for testing. The hearing children used spoken Swedish.

The task used in this study was identical to Meristo et al. (2012). However, looking scores for each child were calculated by subtracting looking time to the incorrect exit from looking time to the correct one, and then dividing it by the total amount of time spent looking at both exits (Senju et al., 2009). The subsequent score ranged from −1 to +1, with scores reliably above zero considered as evidence of correct anticipatory looking.

While hearing children mostly showed correct anticipatory looking (mean differential looking score 0.72), children with implants (*M* = 0.16) and children with hearing aids (*M* = −0.10) performed at chance levels.

All children were also administered three trials of an elicited-response unexpected transfer task (Baron-Cohen et al., 1985). In each trial, they scored 1 point if they correctly answered the test question along with two control questions, yielding a maximum score of 3. Hearing children (*M* = 2.4) and implanted children (*M* = 1.6) performed above chance, while children with hearing aids (*M* = 0.4) did not, and there was a significant correlation between performance on both false belief tasks for the whole sample. Language ability correlated with both spontaneous-response and elicited-response performance.

In addition, all children also completed the Peabody Picture Vocabulary Test to measure their verbal ability. The typically hearing group performed significantly better than the hearing aid group, but did not significantly differ from the cochlear implant group. There was no significant difference between the implant and hearing aids groups, and no significant differences among the groups in non-verbal IQ (Raven’s Coloured Progressive Matrices).

Language ability was significantly correlated with performance on both the spontaneous-response false-belief task and the elicited-response false-belief task for the entire sample. These correlations remained significant even when controlling for chronological age or non-verbal IQ.

The differences between the spontaneous- and elicited-response tasks reveal that while typically hearing children and children with cochlear implants can pass verbally loaded tasks, only typically hearing children perform well on tasks aimed at assessing spontaneous false belief understanding.

Finally, inspired by the previous findings that deaf children from deaf families perform well on elicited response tasks (Meristo et al., 2007), Meristo and Strid (2020) examined the ability of deaf children from deaf families to understand spontaneous false beliefs attribution compared to deaf children with cochlear implants and typically developing hearing children. The study included 46 children aged 1 year 5 months to 8 years 11 months, divided into three groups: 9 profoundly deaf children from profoundly deaf families with a mean age of 4 years 3 months (range 2 years 2 months to 6 years 2 months; all used Estonian Sign Language as their primary and preferred means for communication, no child used cochlear implants), 13 profoundly deaf children with cochlear implants with a mean age of 4 years 9 months (range 1 year 5 months to 8 years 5 months, no children had immediate family members who used sign language), and 24 typically developing hearing children with a mean age of 4 years 5 months (range 1 year 7 months to 8 years 11 months). All participants in the study were without known health issues or additional disabilities that could potentially impact the results.

The task used was a non-verbal eye-tracking false-belief task adapted from Surian and Geraci (2012). The children’s looking times were measured to assess their understanding of a character’s false belief about another character’s location (see Meristo et al., 2016 described above). There were no additional tasks beyond the false-belief task.

The results showed a significant difference in looking times between the three groups. The deaf of deaf children performed significantly better than the implanted children, but there were no differences between the deaf of deaf and the hearing groups. Follow-up *t* tests showed that the deaf of deaf group performed significantly above zero (*M* = 0.47), as did the hearing group (*M* = 0.69). In contrast, the implanted children’s performance did not differ from the chance level of zero (*M* = −0.29). Correlations between chronological age and looking time scores were found only for the implant group, but not for the hearing or the deaf of deaf group. The authors suggest that deaf children from deaf families benefit from early exposure to fluent sign language communication with their caregivers, enabling them to develop false belief understanding comparable to hearing children. In contrast, deaf children with hearing parents have reduced access to such language-based interactions, at least until they receive cochlear implants, which may delay their social-cognitive development. While the study provides intriguing evidence for the role of early language exposure in implicit theory of mind development, the small sample sizes, lack of additional measures, and wide age ranges are limitations that should be addressed in future research. It is also important to note the ongoing replication crisis surrounding spontaneous-response false-belief tasks in infancy research. Many of the seminal findings suggesting infants can attribute false beliefs have failed consistent replication, calling into question the robustness and validity of these results (Kampis et al., 2021; Kulke et al., 2019).

## MAIN THEORETICAL PERSPECTIVES ON THEORY OF MIND DEVELOPMENT

Theoretical perspectives on theory of mind development can be broadly categorized into two main groups: the conceptual change view and the developmental continuity view. Within the conceptual change framework, theories align themselves along a constructivist or an associative learning perspective. Both suggest that theory of mind development is intricately linked to general cognitive processes and learning mechanisms across various domains (Wellman, 2014). These theoretical proposals typically do not credit infants and toddlers with metarepresentational skills. For instance, Cecilia Heyes (2014) introduced the concept of submentalizing to describe the use of domain-general cognitive processes that can mimic mentalizing in social contexts without actually representing mental states. Submentalizing mechanisms enable individuals to navigate social situations and exhibit behaviors that appear to result from mentalizing, without considering the thoughts of others. Examples of the domain-general processes proposed to underlie submentalizing include involuntary attentional orienting, spatial coding of responses, and object-centered spatial coding. Heyes suggests that a broader spectrum of domain-general associative learning processes serves as the framework for the gradual emergence of advanced social cognitive abilities. The cumulative experiences with submentalizing contribute to the understanding of others’ mental states over time, marking a developmental trajectory in social cognition. A conceptual change in theory of mind occurs at around the age of four years (Heyes & Frith, 2014; Wellman, 2014), coinciding with the development of other domain-general abilities such as language and executive function (Heyes, 2018). However, several recent experimental findings run against this view by showing that toddlers’ success in spontaneous response false belief tasks cannot simply be interpreted as false positives due to the effects of domain-general attention and memory limitations (e.g., Király et al., 2023; Surian & Franchin, 2020).

Csibra and Gergely (1998) propose a teleological theory, suggesting that infants possess a naive theory of rational action that allows them to interpret and predict other agents’ goal-directed actions. This theory is based on the idea that actions function to achieve goal-states by the most efficient means available. In this teleological stance, observed behavior is interpreted as an action directed towards a particular end state if it is judged to be the most efficient means available for achieving the goal. Csibra and Gergely argue that mentalistic action explanations, which form an essential component of a mature theory of mind, are conceptually and developmentally derived from this earlier and purely teleological interpretational system present in infancy (Gergely & Csibra, 2003).

Minimalism, as proposed by Ruffman (2023), refers to the theory that infants initially understand only behaviors, rather than mental states or intentions. This theory posits that infants use their inherent statistical learning abilities to detect regularities in behavior, which forms the basis of their understanding of the world around them. Over time, this implicit understanding of behavior evolves into an explicit understanding of mental states. This process is thought to be facilitated by factors such as maternal mental state talk, the child’s evolving understanding of self, and the child’s own language development.

In contrast, the developmental continuity view, often associated with nativist theories, underscores the existence of principles and acquisition mechanisms exclusively dedicated to theory of mind development. This theoretical stance contends that certain cognitive faculties are inherently specialized for understanding and interpreting mental states. Nativist theories propose that theory of mind is either innate or is acquired in the first few months of life (Luo, 2011; Sodian, 2011; Vaish & Woodward, 2005). This perspective is typically construed as implicit, suggesting that individuals automatically and non-verbally process and understand others’ mental states. The nativist perspective is supported by researchers who argue that theory of mind is an evolved, inherent part of human cognition (Baillargeon et al., 2018; Baron-Cohen, 1995; Carey & Spelke, 1996; Carruthers, 2013; Leslie, 1987; Premack, 1990; Surian et al., 2007). This view is supported by studies showing early theory of mind understanding in infants using tasks with minimal processing demands (Burnside et al., 2020). This perspective is also supported by neuroimaging studies showing that young infants can demonstrate understanding of others’ mental states, such as beliefs, desires, and intentions (Hyde et al., 2018; Southgate & Vernetti, 2014). While there is ongoing debate about the exact nature and development of theory of mind, mentalistic theories generally agree that this ability is present early in life and develops rapidly within the first few months.

## ACCOUNTS OF THE IMPACT OF CONVERSATIONAL EXPERIENCE ON THEORY OF MIND DEVELOPMENT

There is now significant evidence suggesting that children with hearing loss who are raised in families where sign language is not the first or native language, and thus do not have the opportunity to engage in fluent conversations with their family members, display a range of deficits in comprehending others’ mental states. These deficits are evident in both traditional and non-traditional tasks (Meristo et al., 2016, 2024), appear early in development (Meristo et al., 2012) and persist well into adulthood (Gagne & Coppola, 2017; Marschark et al., 2019).

Why might restricted access to conversations be linked to poor performance on tasks involving false beliefs? Is there a critical age for acquiring a full-blown theory of mind, as evidence from Nicaraguan deaf children seems to suggest (Morgan & Kegl, 2006)? We argue that there are multiple ways in which language experience can plausibly affect theory of mind. Here we will focus on five possible routes that can provide an explanation for why deaf children from hearing families show difficulties in theory of mind development.

One theory, which we will call the *pragmatic account*, suggests that social factors help children, including deaf children, to respond to direct questions in traditional false-belief tasks (Westra & Carruthers, 2017; Yazdi et al., 2006). In a typical *unexpected transfer* false-belief scenario, a character places an object in one location, and the object is later moved to another location without the character’s knowledge. Children are then asked a direct question, such as *Where will the character look for the object?* Pragmatic factors might lead children to misinterpret the question, causing them to answer based on where the character should look or where the object actually is (Surian & Leslie, 1999). The restricted access to regular engagement in social interactions involving discussions of mental states could make it difficult for deaf children to overcome this pragmatic ambiguity, not allowing them to correctly interpret the question and provide the appropriate response. The pragmatic account can explain difficulties on elicited-response verbal tasks, but cannot explain difficulties on nonverbal tasks, that is tasks that do not involve explicit verbal questions. However, there is evidence that deaf children do not perform well on these tasks (Meristo et al., 2012, 2024).

Another possible account is that conversational experience triggers the activation of an innate mentalizing competence. This *triggering account* is consistent with evidence from studies showing that deaf toddlers face difficulties on theory of mind tasks. However, it is somewhat at odds with results from typically hearing infants reporting mentalist attributions in the first year (e.g., Kovács et al., 2010), well before they can engage in any conversation. The plausibility of this account therefore rests on the interpretation of these results and their implications for the attribution of very early competences that, at present, remains controversial (Baillargeon et al., 2018; Phillips et al., 2015).

A third possibility, which could be called the *practice account*, is that the association between social interaction and children’s performance in false belief tasks extends beyond answering direct questions about mistaken agents’ actions. In the typical false belief situation, a variety of processes aid children in determining where a mistaken agent will search for an object. These processes involve focusing on the agent and the mental-state information in the scene, and utilizing this data to deduce the agent’s mental states (Roby & Scott, 2016). When the agent reappears, children must recall the agent’s mental states and use them to anticipate their actions. These processes are crucial regardless of the method used to assess children’s understanding, be it through responses to direct questions or anticipatory looking. Children’s social experiences might have an impact on each of these processes. Conversations could help children connect specific behaviors or situational cues to particular mental states, enhancing their ability to infer others’ mental states and predict their actions in a variety of situations. Using mental-state language in conversations offers structured practice in remembering events and the mental states of those involved, providing children with a valuable exercise in working memory mechanisms involved in retrieving and retaining belief-relevant information (Marschark et al., 2019; San Juan & Astington, 2012). Consequently, social experiences could bolster children’s ability to retrieve mental-state relevant information when required. This explanation suggests that access to language-based interaction plays an important role for the expression of false belief abilities in deaf children, rather than its emergence. This perspective aligns with the developmental continuity view, which posits a consistent progression in the development of mentalizing skills.

The fourth possibility, proposed by the *constructivist account*, aligns with the conceptual change view and suggests that participation in conversation and social interaction are instrumental in the emergence of the capacity to represent beliefs. These activities equip children with the necessary linguistic and social experiences to construct the concept of belief and comprehend the idea that beliefs can be incorrect and that perspectives can vary. Conversations offer a stage for children to express their mental content publicly, which then becomes a focal point of group attention. This collective focus facilitates the exploration of various viewpoints, including the realization that perspectives can vary and may not correspond directly to the actual situation (Tomasello, 2018). This process necessitates the child’s ability to juggle different perspectives, including an objective one—a skill that starts to develop in children around the ages of 4 to 5 years. In this context, infants’ and toddlers’ success in various non-traditional tasks does not necessarily reflect their understanding of false beliefs. Instead, it may simply mirror results from tracking behavioral patterns (Ruffman, 2023), responses to perceptual cues (Heyes, 2014), or basic mental states other than beliefs (Butterfill & Apperly, 2013). Yet, recent work on infants’ ToM reported results that are difficult to explain away as the outcome of these alternative explanations (e.g., Király et al., 2023). Moreover, to strengthen the constructivist account, future research will need to assess whether the outcomes of training studies (e.g., interventions involving children with autism) are due to the acquisition of core theory of mind or rather the acquisition of alternative non-mentalistic strategies (Wellman et al., 2002).

All four possible accounts considered above focus on the role of experience deprivation in theory of mind development. It is worth emphasizing that these accounts are not mutually exclusive, as theory of mind competence is multifaceted. One can hold a nativist stance for some components (e.g., goal attribution and action prediction based on teleological reasoning) while supporting a conceptual change view for others (e.g., metarepresentational reasoning; Spelke, 2022).

Finally, we would like to mention a fifth possible account that shifts the focus from experience deprivation to atypical neural maturation. This neural account is inspired by recent results from studies of congenitally blind children, a population that has been found to perform poorly on a variety of theory of mind tasks (Hobson & Bishop, 2003). These findings were initially used to support explanations of theory of mind development as linked to visual experience and communication. However, Begeer et al. (2014) found that not all congenitally blind children show a delay in theory of mind development: these difficulties were observed in children whose blindness was due to neural abnormalities (e.g., retinopathy, damage to the optic tract), but not in those whose blindness resulted from non-neural abnormalities of the ocular bulb. This suggests that theory of mind delays in congenitally blind children cannot be explained solely by reference to experience deprivation, since deprivation was similar in the two groups. No previous study on deaf children’s theory of mind delay has tackled this problem or attempted to disentangle the effect of experience from the independent role of the neural bases of deafness. Thus, further research is needed to assess the viability of the neural account.

## INTERVENTIONS TO IMPROVE THEORY OF MIND SKILLS IN CHILDREN WITH HEARING LOSS

The evidence reviewed above consistently points out that children with hearing loss, particularly those who have been raised by hearing parents, show difficulties in acquiring theory of mind skills. Fortunately, research also suggests that the impact of deafness on the development of cognitive skills can be mitigated by providing early access to language (e.g., Hall et al., 2017, 2018). Moreover, the acquisition of theory of mind skills can be facilitated by the implementation of appropriate training programs (e.g., Wellman & Peterson, 2013).

Hall et al. (2017) found that deaf native signers perform like hearing controls in a number of measures of executive functions skills. This result is in stark contrast with the performance obtained from non-native signers in previous studies, which found that non-native signers perform significantly worse on EF measures compared to hearing controls (e.g., Hintermair, 2013; Kronenberger et al., 2014). Converging findings were also reported in Hall et al. (2018) by comparing hearing children with native signer deaf children as well as deaf children with cochlear implant. These results provide support for the critical role of language for executive functions in deaf children. They also suggest that deprivation of acoustic signals per se plays, if any, a marginal role. Given the involvement of executive functions skills in theory of mind reasoning, these results also emphasize the need to minimize the language deprivation experienced by deaf children living with hearing parents.

Initial evidence on the effectiveness of a training program to facilitate the development of theory of mind skills in deaf children was reported by Wellman and Peterson (2013), who tested a group of 43 deaf children of hearing parents aged 5 to 13 years (a related intervention has been outlined also by Morgan, 2015). The materials and procedures in this study were based on the thought bubbles program devised for an intervention on children with autism (Wellman et al., 2002). This 6-week intervention produced reliable improvements in the performance on standard ToM tasks in all participants. On average, children in the theory of mind training group improved by nearly one full point on the 3-point false belief measure (from a mean of 1.15 at pretest to 2.08 at posttest) and increased by more than 1.3 steps on the 5-step theory of mind scale (from 2.23 at pretest to 3.62 at posttest). This represents dramatic progress compared to the control groups, who showed little to no improvement.

Another study has explored the effects of training on sentential complements and its influence on theory of mind development (Tucci et al., 2016). In this study, 13 American children with hearing loss (*M* = 5 years 2 months), participated in a 15- to 18-week training program focusing on of the specific linguistic structure of sentential complements. Previous research found a correlation between the acquisition of sentential complements and theory of mind understanding in typically developing hearing children as well as in children with hearing loss (de Villiers & de Villiers, 2012). Six children attended total communication (pre)kindergarten, and 7 used spoken language in classroom education. The paper does not provide any information about the children’s hearing aids, levels of hearing loss or parents’ and siblings’ sign language skills. The training focused on the relationship between the main verb of a sentence (e.g., ‘She thinks …’) and the complement structure that completes the sentence (e.g., ‘She thinks that it is a shoe.’), adapted from Wellman and Peterson (2013). The results showed that the children demonstrated improved understanding of theory of mind post-training. That is, while all children failed the pre-test unexpected transfer task, 8 out of 12 passed the test at posttest stage. While the average change for the posttest measure was only 0.33 stages, the study still indicates the potential benefits of training children on grammatical structures with sentential complementation.

In a more recent study, Durrleman et al. (2022) replicated these findings with 21 French children with mild to moderate hearing loss, aged 8 years and 11 months (range 5 years 11 months to 11 years 2 months). Sixteen of the children used cochlear implants and 5 wore conventional hearing aids, all had hearing parents. The study involved testing children with hearing loss before and after a 6- to 8-week training program to assess the effects on their understanding of complements and theory of mind. The training program included activities of varying degrees of difficulty, all targeting complements of verbs of communication. The results indicated improvements in both complements and theory of mind tasks after the training, suggesting that the practice with sentential complements may enhance theory of mind reasoning in children with hearing loss.

Another recent study found that a short training on theory of mind had a positive effect on the theory of mind skills of college students with hearing loss (Wu et al., 2022). College students lag behind hearing controls in the development of theory of mind skills (Marschark et al., 2019). Wu et al. (2022) adapted a theory of mind intervention developed by Cavallini et al. (2015; see also Bianco et al., 2019). The training consisted of four sessions in which participants were encouraged to reflect on the mental states of some story characters. Significant improvements were recorded both at immediate and at delayed (1 month) posttests. Improvements were found not only on skills demonstrated in theory of mind stories tasks, but also in the ability to reflect in a mentalistic way when watching silent animations involving geometrical shapes (as in Castelli et al., 2000). This outcome suggests a good level of generalization to a variety of situations. No improvements in theory of mind skills were observed in a control group that was enrolled in a training in which the conversations were focused on physical rather than mental processes. A further study on deaf adolescents reported positive effects of theory of mind training on subjective measures of social-emotional functioning, but not on objective measures of theory of mind skills (Smit et al., 2024).

Overall, research on training interventions for children with hearing loss yielded promising results, but the available evidence is still scarce and further work is needed to consolidate and extend these initial findings.

## CONCLUSIONS

There are several pathways through which language and conversational experience may significantly affect the development of theory of mind. We highlighted the possible effects on pragmatics (i.e., facilitation in responding to direct questions), triggering (i.e., the activation of mental structure, a process or a representational resource), exercise and automatization (i.e., the repeated use of a process that will make its use less costly and quicker), and conceptual change (i.e., the revision of central concepts in the domain of mental state knowledge). As mentioned above, these accounts are not necessarily mutually exclusive. Some of them may well prove to be the best explanation for part of the findings, but perhaps not for all of them. The evidence from children with hearing loss reviewed here curbs this debate, but a conclusive answer requires a deeper understanding of the processes underlying infants’ success and failures in spontaneous response mentalizing tasks.

Deaf children, including deaf children raised by hearing parents, may succeed in spontaneous communication and the ability to develop social relationships, which sets them apart from most autistic children—despite the similarities in performance of the two populations on false belief tasks. This suggests that failures in false belief tasks should be interpreted with caution when trying to estimate children’s overall competence in social cognition (Begby, 2023). Peterson and Wellman (2019) found that 5-to-12-year-old deaf children, raised by hearing parents, were able to generate good mentalistic explanations of agents that acted consistently with their false beliefs, despite failing standard false belief tasks. This should prompt the use, in future works, of a greater variety of tasks and methods, including tasks assessing children’s pragmatic skills (Surian et al., 2010) or methods yielding a quantification of their strengths and weaknesses in daily social interactions (Vissers & Hermans, 2018). These studies will reveal more details about the similarities, but also about the important differences in how social cognition is acquired by deaf children and other populations with theory of mind difficulties, such as autistic individuals.

While there may be differences in theory of mind development between children with and without hearing loss, early intervention and a supportive environment can play a crucial role in bridging this gap. The performance of deaf children with cochlear implants (Meristo et al., 2016; Peters et al., 2021) suggests that early implantation might be an effective way to reduce or avoid delay in theory of mind development. However, findings are not consistent enough to show that this strategy will reliably lead to the desired developmental outcomes. Other important implications for practitioners concern educational settings and activities. One study showed that the type of communication chosen in education affects the development of theory of mind, with native deaf children attending bilingual (sign-plus-speech) centers outperforming deaf children attending oral-only primary school (Meristo et al., 2007). Training studies have repeatedly reported reliable improvements obtained by simple interventions involving reading books with thought bubbles or other activities that prompt people to reflect and talk about the mental states of story characters or participants. Activities such as reading books (Chilton, 2017; Kidd & Castano, 2013), pretend play, and using language rich in mental states terms proved to be beneficial for the development of theory of mind skills in deaf children and adults. Finally, practitioners could inform parents that overhearing is viewed as a crucial factor in the development of theory of mind skills. While hearing aids and cochlear implants are valuable, they cannot fully replicate the overhearing potential of a typically hearing ear. Therefore, using language rich in mental states terms is an important tool to provide everyday opportunities for children with hearing loss not only to understand the meanings of these words, but also to practice their abilities to relate to others’ perspectives.

## FUNDING INFORMATION

This study was supported by the Swedish Research Council for Health, Working Life and Welfare to M. Meristo (2021-01858) and by the Ministero dell’Universitá e della Ricerca to L. Surian (PRIN-PNRR grant no. P2022M9EKK).

## AUTHOR CONTRIBUTIONS

M. M.: Conceptualization; Writing – original draft; Writing – review & editing. L. S.: Conceptualization; Writing – review & editing.
